# Blood RNA analysis can increase clinical diagnostic rate and resolve variants of uncertain significance

**DOI:** 10.1038/s41436-020-0766-9

**Published:** 2020-03-03

**Authors:** Htoo A. Wai, Jenny Lord, Matthew Lyon, Adam Gunning, Hugh Kelly, Penelope Cibin, Eleanor G. Seaby, Kerry Spiers-Fitzgerald, Jed Lye, Sian Ellard, N. Simon Thomas, David J. Bunyan, Andrew G. L. Douglas, Diana Baralle, Splicing and disease working group, Swati Naik, Nicola Ragge, Helen Cox, Jenny Morton, Mary O’Driscoll, Derek Lim, Deborah Osio, Frances Elmslie, Camilla Huber, Julie Hewitt, Heidy Brandon, Meriel McEntagart, Sahar Mansour, Nayana Lahiri, Esther Dempsey, Merrie Manalo, Tessa Homfray, Anand Saggar, Jin Li, Julian Barwell, Kate Chandler, Tracy Briggs, Sofia Douzgou, Julian Adlard, Alison Kraus, Sarju Mehta, Amy Watford, Alan Donaldson, Karen Low, Gabriela Jones, Abhijit Dixit, Elizabeth King, Nora Shannon, Marios Kaliakatsos, Merrie Manalo, Shelagh Joss, Meena Balasubramanian, Diana Johnson, Sarah Everest, Claire Salter, Victoria Harrison, Gillian Wise, Audrey Torokwa, Victoria Sands, Esther Pyle, Tessy Thomas, Katherine Lachlan, Nicola Foulds, Andrew Lotery, Andrew Douglas, Simon Hammans, Emily Pond, Rachel Horton, Mira Kharbanda, David Hunt, Charlene Thomas, Lucy Side, Catherine Willis, Stephanie Greville-Heygate, Rebecca Mawby, Catherine Mercer, Karen Temple, Esther Kinning, Ognjen Bojovic, L. Archer

**Affiliations:** 1 Human Development and Health, Faculty of Medicine, University of Southampton, Southampton, UK; 2 Wessex Regional Genetics Laboratory, Salisbury District Hospital, Salisbury, UK; 3 Exeter Genomics Laboratory, Royal Devon & Exeter NHS Foundation Trust, Exeter, UK; 4 Translational Genomics Unit, Broad Institute of MIT and Harvard, Cambridge, MA USA; 5 Wessex Clinical Genetics Service, University Hospital Southampton NHS Foundation Trust, Southampton, UK

**Keywords:** RNA splicing, variant interpretation, genetic diagnosis, genomic medicine, RNA-seq

## Abstract

**Purpose:**

Diagnosis of genetic disorders is hampered by large numbers of variants of uncertain significance (VUSs) identified through next-generation sequencing. Many such variants may disrupt normal RNA splicing. We examined effects on splicing of a large cohort of clinically identified variants and compared performance of bioinformatic splicing prediction tools commonly used in diagnostic laboratories.

**Methods:**

Two hundred fifty-seven variants (coding and noncoding) were referred for analysis across three laboratories. Blood RNA samples underwent targeted reverse transcription polymerase chain reaction (RT-PCR) analysis with Sanger sequencing of PCR products and agarose gel electrophoresis. Seventeen samples also underwent transcriptome-wide RNA sequencing with targeted splicing analysis based on Sashimi plot visualization. Bioinformatic splicing predictions were obtained using Alamut, HSF 3.1, and SpliceAI software.

**Results:**

Eighty-five variants (33%) were associated with abnormal splicing. The most frequent abnormality was upstream exon skipping (39/85 variants), which was most often associated with splice donor region variants. SpliceAI had greatest accuracy in predicting splicing abnormalities (0.91) and outperformed other tools in sensitivity and specificity.

**Conclusion:**

Splicing analysis of blood RNA identifies diagnostically important splicing abnormalities and clarifies functional effects of a significant proportion of VUSs. Bioinformatic predictions are improving but still make significant errors. RNA analysis should therefore be routinely considered in genetic disease diagnostics.

## INTRODUCTION

Use of next-generation sequencing (NGS) technologies in clinical practice has led to an unprecedented increase in the number of variants being identified in patients undergoing investigation for genetic disorders. Incomplete knowledge of the functional effects of variants and our limited understanding of genotype–phenotype correlations severely compromises attempts to definitively assign or refute pathogenicity for a large proportion of variants. Variant of uncertain significance (VUS) reporting rates vary over time and depending on local reporting policies but of all variants listed on ClinVar (as of 13 November 2019), 48% are asserted to be of uncertain significance (Figure S1).^1^ In a clinical setting, this uncertainty has major implications for patients and their families, where having a clear genetic diagnosis can allow evidence-based management decisions to be taken and informed reproductive choices to be made.^2,3^

RNA splicing is thought to be disrupted by up to 62% of all pathogenic single-nucleotide variants (SNVs).^4^ Current bioinformatic filtering strategies and clinical interpretation guidelines tend to focus heavily on amino acid–level effects in terms of both variant detection and assignment of pathogenicity.^5^ This can lead to synonymous variants being filtered out at an early stage of analysis, even though such variants may affect splicing. Similarly, although deep intronic variant data are increasingly available via NGS approaches like genome sequencing, such noncoding variants are rarely considered owing to a lack of evidence on which to base interpretations. Where bioinformatic predictions suggest that a variant affects splicing, there can be scope for additional RNA-based investigations. However, such splicing prediction tools frequently produce conflicting results and their accuracy and utility decreases outside of canonical splice sites and consensus splice regions.^6^

In this study, we looked for RNA splicing defects in a large cohort of VUSs identified in patients who had undergone diagnostic genetic testing. We compare in silico predictions of splicing with the results of blood RNA analysis and provide examples that illustrate the clinical utility of RNA-based testing in clinical diagnostics. These results support the routine use of RNA analysis in clinical diagnostic practice.

## MATERIALS AND METHODS

### Patients and variants

A cohort of patients with VUSs identified through routine diagnostic genetic testing was identified primarily through the Wessex Regional Genetics Laboratory, Salisbury (203 variants), with seven other patients identified through the Exeter Genomics Laboratory. Additional patients with 47 variants from across the UK were identified through the Splicing and Disease research study at the University of Southampton, ethically approved by the Health Research Authority (IRAS Project ID 49685, REC 11/SC/0269) and by the University of Southampton (ERGO ID 23056). Informed consent for splicing studies was provided for all patients from whom samples were obtained.

### RNA extraction and cDNA preparation

Blood was collected in PAXgene Blood RNA tubes and RNA extracted using the PAXgene Blood RNA Kit (PreAnalytiX, Switzerland). Complementary DNA (cDNA) was synthesized via reverse transcription using random hexamer primers. For details of each laboratory’s individual protocols, see Supplementary Methods.

### Reverse transcription polymerase chain reaction (RT-PCR) analysis

Primers were designed to amplify the region around each variant (sequences available on request). Wherever possible, primer sequences were positioned at least two exons up- and downstream of the target variant. PCR products were evaluated by agarose gel electrophoresis against control samples and purified PCR products were analyzed by direct Sanger sequencing. In a number of cases, PCR products separated by gel electrophoresis were purified and sequenced or cloned into *E. coli* using a TA-cloning vector. Plasmids recovered from single-clone bacterial cultures were analyzed by Sanger sequencing. Please see Supplementary Methods for laboratory-specific PCR, Sanger sequencing, and bacterial cloning conditions.

### RNA-seq analysis

For full information, see Supplementary Methods. In brief, selected RNA samples underwent RNA-seq via Novogene (Hong Kong) using the NEBNext Globin and rRNA Depletion Kit and NEBNext Ultra Directional RNA Library Prep Kit for Illumina (New England Biolabs, MA). At least 70 M 150-bp paired-end reads (21 Gb raw data) per sample were generated on a HiSeq 2000 instrument (Illumina, CA). Raw data were filtered for quality and had adapter sequences removed by Novogene. Reads were aligned to the human genome (GRCh38) using STAR (v2.6.1c)^7^ on the University of Southampton’s IRIDIS 4 high performance computing cluster and the splicing effects of specific variants was assessed visually using the Integrative Genomics Viewer^8^ (Broad Institute, MA) and its inbuilt Sashimi plot function.^9^ A threshold of three or more reads was required to call an abnormal splice event and use of the novel junction had to reach at least 5% read support compared with the alternative canonical junction. Where appropriate, percent spliced in (PSI) values were calculated for abnormal splicing events.^10^

### In silico splicing predictions

All variants were assessed bioinformatically for predicted splicing effects using Alamut Visual version 2.11 (Interactive Biosoftware, Rouen, France), which incorporates predictions from MaxEntScan (MES), NNSplice, and Splice Site Finder (SSF).^11–13^ Individual tools were deemed to predict altered splicing where the change in splice site score was ≥10% (MES) or ≥5% (NNSplice and SSF).^14,15^ An overall prediction of altered splicing was called where two of three Alamut programs agreed. Additional splice prediction information was obtained using Human Splicing Finder (HSF) version 3.1 (threshold ≥ 0.2) and from publicly available SpliceAI scores (v1.3) for variants (threshold ≥ 0.2).^16,17^ Missing score rate, sensitivity, specificity, overall accuracy, and positive and negative predictive values were calculated for each tool individually and for the combined Alamut 2/3 assessment (equations in Supplementary Methods). The package pROC (v1.15.3)^18^ was used in R (v3.5.1)^19^ in RStudio^20^ to plot receiver operating characteristic (ROC) curves (ggplot2, v3.2.1)^21^ for the overlapping set of variants scored by all tools and calculate the area under the curve (AUC) for each tool and for the combined Alamut 2/3 assessment.

## RESULTS

### Variants affecting splicing

**Fig. 1:**
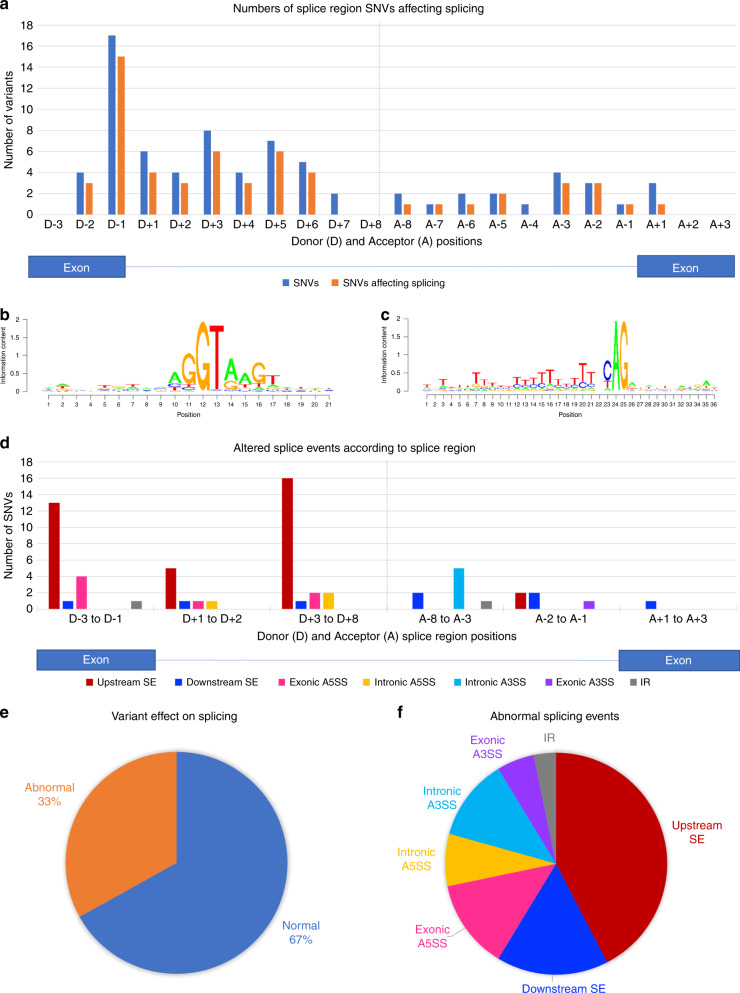
Variant locations and effects on splicing. (**a**) Plot of the numbers of single-nucleotide variants (SNVs) in this cohort (multinucleotide variants not included) present at each donor (D-3 to D+8) and acceptor (A-8 to A+3) splice region position, along with the numbers of these found to affect splicing. (**b**, **c**) Position–weight matrices of nucleotide sequence across the splice donor (**b**) and acceptor (**c**) regions as determined for the specific exon–intron junctions analyzed in this study. In this representation, the donor splice site +1 position correlates to position 12 in (**b**), while the acceptor splice site −1 position correlates to position 25 in (**c**). (**d**) Abnormal splicing effects plotted by SNV location. Sequence ontology defines the donor splice region as extending from the third last nucleotide of the exon (D-3) to the eighth nucleotide of the intron (D+8) and the acceptor splice region as extending form the eighth last nucleotide of the intron (A-8) to the third nucleotide of the exon (A+3).^22^ (**e**) Overall proportion of all variants affecting splicing in this cohort. (**f**) Proportions of different abnormal splicing events identified in this cohort. *A3SS* alternative 3´ splice site; *A5SS* alternative 5´ splice site*, IR* intron retention, *SE* skipped exon.

A total of 257 different variants were assessed for their effect on splicing (Table S1). Two-hundred forty-three variants were single-nucleotide substitutions, while 14 variants spanned multiple nucleotides (10 deletions, 1 insertion, 2 deletion–insertions, and 1 deletion with an in *cis* SNV). Variants were located across 62 genes in total, with particularly high numbers of variants in *BRCA1* (42), *BRCA2* (42), and *FBN1* (87). In all, 85 variants (33%) were found to be associated with abnormal splicing. Of 57 single-nucleotide substitution variants, 44 (77%) located within the donor splice site or splice region (defined by sequence ontology as extending from the third last base of the exon up to the eighth base of the intron) and 13/19 single-nucleotide substitution variants (68%) located within the acceptor splice site or splice region (from the eighth last base of the intron up to the third base of the exon) were found to alter splicing (Fig. 1).^22^ One hundred seventy-five variants in total did not involve annotated splice regions and of these, 23 (13%) affected splicing (21/167 single-nucleotide substitutions).

Thirty-nine variants were associated with skipping of the upstream exon (as defined by the location of a variant lying closer to that exon’s donor splice site than to an acceptor splice site), which was the most frequent splicing abnormality identified. Only 15 variants were associated with downstream exon skipping; however, the analyzed variant cohort contained relatively fewer acceptor region variants. These exon skipping figures include three cases in which both upstream and downstream exons were skipped and one case of double upstream exon skipping. Twenty-three variants led to use of an alternative splice donor site and 16 to use of an alternative splice acceptor site, while intron retention was associated with only three variants. For four variants there were multiple splicing abnormalities identified.

### Illustrative examples

**Fig. 2:**
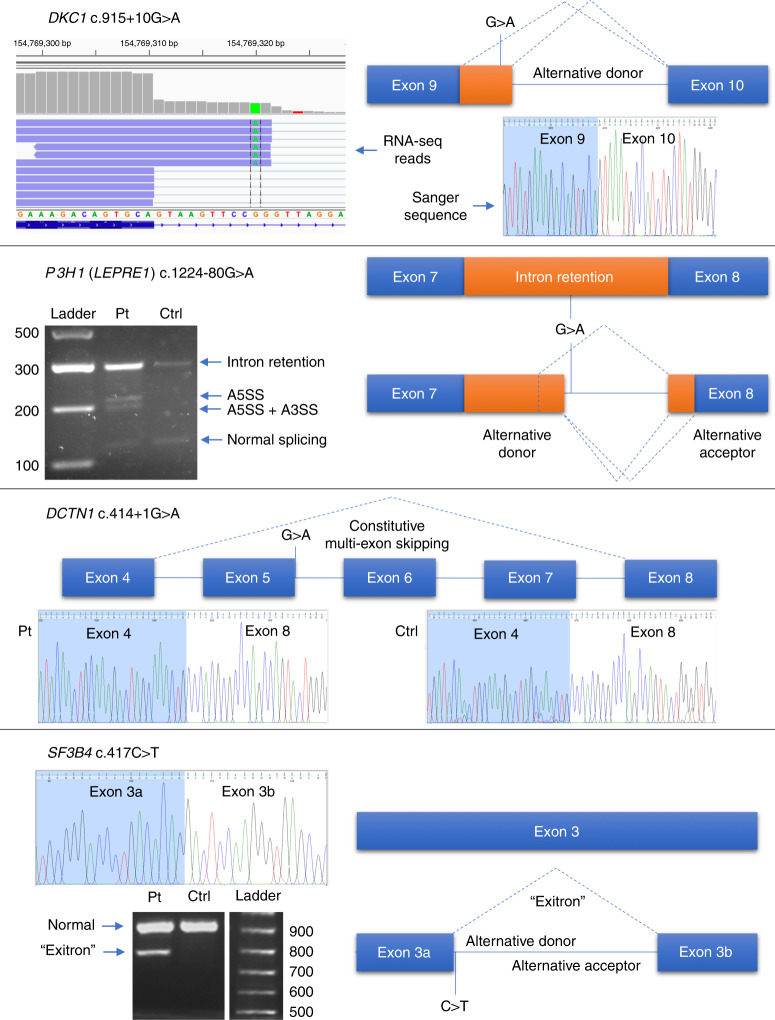
Illustrative examples of variant splicing analysis. *DKC1* c.915+10G>A could not be identified by reverse transcription polymerase chain reaction (RT-PCR) and Sanger sequencing but alternative donor splice site usage was identified by RNA-seq. *P3H1* (*LEPRE1*) c.1224-80G>A causes at least three abnormal splicing events using alternative splice donor and acceptor sites, as well as increasing levels of intron retention. *DCTN1* c.414+1G>A appears to alter a canonical splice donor site but exons 5–7, although annotated, are never expressed and are constitutively spliced out. *SF3B4* c.417C>T is a synonymous coding variant but causes formation of a 125-nt “exitron,” an intronic region within an exon. *A3SS* alternative 3´splice site, *A5SS* alternative 5´ splice site, *Ctrl* control, *Pt* patient.

Several examples from this cohort are pertinent in illustrating the variability in splicing effect seen across different variants (see Fig. 2).

#### RNA-seq detects a splice variant missed by Sanger sequencing

This hemizygous *DKC1* c.915+10G>A variant, identified in a male patient with dyskeratosis congenita, produced normal results from direct Sanger sequencing of RT-PCR products (Fig. 2). Similarly, gel electrophoresis did not suggest the presence of more than one RT-PCR product. However, RNA-seq revealed creation of a novel intronic donor splice site, resulting in an insertion of 11 extra nucleotides, which was subsequently confirmed by isoform-specific RT-PCR and sequencing of cloned amplicons. The novel junction had a PSI value of 20%, calculated as the number of length-normalized inclusion reads divided by the total number of length-normalized inclusion and exclusion reads.^10^

#### A complex deep intronic variant affects splicing

This heterozygous *P3H1* (*LEPRE1*) c.1224-80G>A variant was identified in a patient with osteogenesis imperfecta. RT-PCR revealed a variety of differently sized bands on electrophoresis and PCR product cloning identified at least four alternative splicing events, including intron retention, creation of two novel intronic splice donor sites (inserting 68 or 92 nt), with some additional use of an alternative exonic splice acceptor site (inserting 92 nt intronic sequence but deleting the first 17 nt of exon 8). RNA-seq analysis was only able to confidently identify use of one of the two intronic splice donor sites. Interestingly, the amino acid sequence of any intron retention event (including those utilizing the subsequent novel intronic donor site) is predicted to result in introduction of a premature termination codon immediately beyond the end of exon 7.

#### An apparent canonical splice site variant has no consequence

A heterozygous canonical splice donor site *DCTN1* c.414+1G>A variant in intron 5 was predicted to disrupt splicing based on NM_004082.4. However, the variant was found to be present at a relatively high minor allele frequency (MAF) of 3.0 × 10^−4^ in the Latino population and 6.4 × 10^−5^ in the gnomAD database (rs576198476). RT-PCR analysis identified that *DCTN1* exons 5–7 are in fact constitutively skipped in both this patient and in controls, negating any potential splicing effects caused by the variant.

#### A deep exonic cryptic splice site

This heterozygous *BRCA1* c.4868C>G transversion 119 nt upstream from the donor splice site of *BRCA1* exon 15 is predicted to introduce a conservative alanine to glycine substitution at amino acid 1623. However, RNA analysis shows that this variant in fact creates an exonic cryptic splice donor site at this position, leading to a 119-nt deletion and frameshift of the transcript.

#### A “likely benign” intronic variant causes pathogenic exon skipping

A heterozygous noncoding *BRCA1* c.5153-26A>G transition 26 nt upstream from the start of exon 18 is annotated as “likely benign” on ClinVar (rs80358109). However, RNA analysis confirms that this variant induces skipping of the downstream exon 18, resulting in an out-of-frame transcript. Interestingly, although there is no predicted effect on the native splice acceptor site, several prediction tools incorrectly suggest creation of a novel cryptic acceptor site.

#### A deep intraexonic splice effect

A heterozygous *SF3B4* c.417C>T synonymous variant located 254 nt into exon 3 was predicted to lead to an enhanced alternative splice site. RT-PCR analysis confirmed the creation of an alternative deep exonic splice donor site. However, use of this novel donor site was found to be coupled to use of a novel splice acceptor site also within *SF3B4* exon 3, leading to an intraexonic deletion of 125 nt. This effect has previously been reported for this variant using a minigene assay.^23^

### RNA-seq coverage

Seventeen samples also underwent RNA-seq analysis. In four cases, RNA-seq was able to detect a splicing abnormality consistent with initial RT-PCR results. In one case (*DKC1*), RNA-seq identified a splicing abnormality not initially detected by RT-PCR. In another case (*SF3B4*), the splicing abnormality seen by RT-PCR was only seen in two RNA-seq reads and so fell below the reporting threshold. In 11 other cases, no reportable splicing abnormality was detected. Of note, splice junction depth of coverage varied considerably across assayed genes and also within genes, which in several cases limited the ability of RNA-seq to detect low-level splice junction usage.

### Bioinformatic splicing predictions

**Fig. 3:**
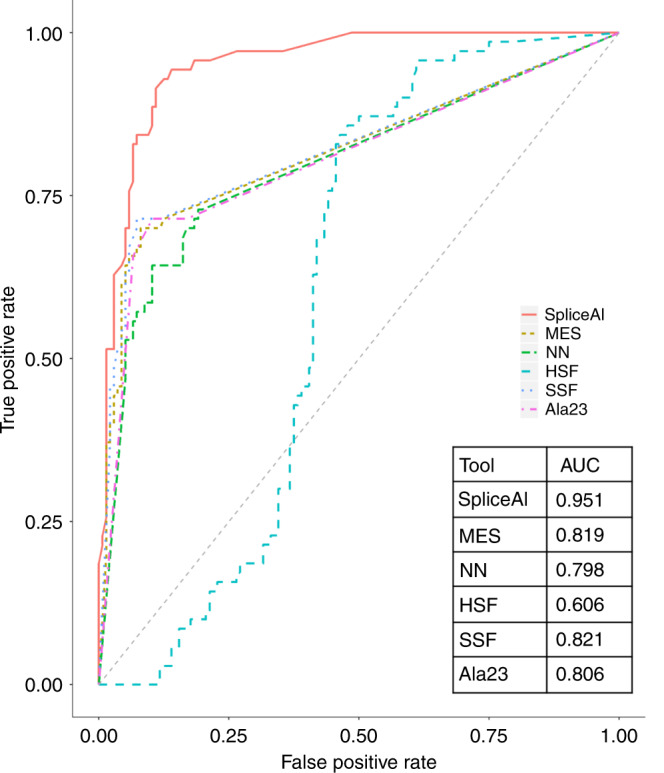
Bioinformatic tools for predicting abnormal splicing. Receiver operating characteristic (ROC) curves and area under the curve (AUC) comparing in silico methods for predicting splice disruption in overlapping set of experimentally validated variants scored by all measures (136 non–splice disrupting, 70 splice disrupting). *HSF* human splicing finder, *MES* MaxEntScan (Alamut), *NN* NNSplice (Alamut), *SSF* SpliceSiteFinder (Alamut). Ala23 = number of Alamut tools exceeding specified thresholds.

We scored all variants with Alamut Visual (v2.11), including MES, NNSplice, and SSF, and also with HSF and SpliceAI. Thresholds for change were selected above which a variant was deemed to be predicted to be splice affecting based on previous literature.^14,15,17^ A combined Alamut score was also calculated, where a variant was deemed to be predicted as splice affecting if two of three individual tools within Alamut passed the defined threshold. The overall sensitivity, specificity, accuracy, and positive and negative predictive values for each tool and the combined Alamut assessment are given in Table 1, based on all variants that were scored by each method. Figure 3 shows ROC curves with AUC values based on the overlapping set of variants scored by all tools. SpliceAI performed the best in predictions of splicing disruption of all the tools/approaches across all of the considered metrics, with overall accuracy exceeding 90% (see Table 1).

**Table 1:** Performance assessment of in silico prediction tools on experimentally validated variants (*n* = 257).

Scoring metric	*n* Missing	Sensitivity	Specificity	Accuracy	PPV	NPV
HSF (2%)	28	0.8941	0.3958	0.5808	0.4663	0.8636
SpliceAI (0.2)	11	0.8987	0.9162	0.9106	0.8353	0.9503
Alamut SSF (5%)	5	0.7317	0.9294	0.8651	0.8333	0.8778
Alamut MES (10%)	1	0.7381	0.9070	0.8516	0.7949	0.8764
Alamut NNSplice (5%)	11	0.6923	0.8631	0.8089	0.7013	0.8580
Alamut 2/3	14^a^	0.7237	0.9162	0.8560	0.7971	0.8793

Values have been calculated omitting the missing scores for each tool.

*HSF* Human Splicing Finder, *MES* MaxEntScan, *NPV* negative predictive value, *PPV* positive predictive value, *SSF* Splice Site Finder.

^a^11 variants missing one score, three variants missing two scores.

## DISCUSSION

### VUS clarification and clinical impact through splicing analysis

This study has helped to clarify the effects on splicing of over 250 VUSs in clinically important disease genes. Thirty-four percent of these VUSs were found to affect splicing. However, while this overall figure is certainly within the range of previous estimates for the proportion of variants affecting splicing, it should be noted that this cohort of variants was specifically selected for splicing studies. As such, there will have been some intrinsic bias in selection, since we expect variants were more likely to be referred for RNA studies if they fell within a splice region or if clinical diagnostic laboratories had already highlighted a potential predicted effect on splicing. Furthermore, the prior probability of these patients having a pathogenic variant in the genes tested is likely to be increased, since UK diagnostic genetic testing generally requires that a patient’s phenotype potentially fits with the genes being tested. Nevertheless, this cohort does represent a true-to-life set of clinically identified VUSs for which clarification of pathogenicity was sought by referring clinicians.

The results of this study show that RNA splicing analysis, using RT-PCR or transcriptomics, has the ability to produce clear results that help clarify variant interpretation. Where abnormal splicing is detected, this analysis constitutes a functional assay that provides supporting evidence of pathogenicity.^5^ In many such cases, these results therefore have direct clinical utility by allowing a genetic diagnosis to be made. Indeed, the results of at least one of these cases (*AARS*) has been used to inform prenatal testing in a subsequent pregnancy.

Only 30% of the variants in this study fell within annotated splice regions, while 13% of non–splice region variants still affected splicing. This highlights the need to consider possible splicing effects whenever deep exonic or deep intronic variants are identified. With increasing use of genome sequencing, increasing numbers of intronic variants will be identified through clinical diagnostic testing. Interpretation of such variants beyond the splice region remains largely uncertain. However, through RNA analysis, potential splicing effects of such variants can be detected.

Furthermore, a number of these results illustrate the danger of assuming the effects of splice site variants. The *DCTN1* c.414+1G>A example is a case in point of a benign canonical splice site variant and our cohort also includes two normal *BRCA2* canonical splice site variants (*BRCA2* c.6937+1G>T and *BRCA2* c.8331+2T>C) that do not appear to cause abnormal splicing (with the caveat that splicing effects in blood may potentially differ from those in other tissues). In addition, the *SF3B4* example shows how difficult it can be to predict splice junction usage, since even if one correctly identifies creation of a cryptic donor site, one may not necessarily predict the acceptor site it will use. This particular variant appears to create a type of noncanonical splicing event known as an “exitron,” where a novel intron is defined entirely within a large exon.^24^

### Targeted testing and transcriptome-wide analysis

Our analysis helps provide some insight into the comparative use of RT-PCR and RNA-seq to look at splicing. Compared with transcriptome-wide RNA-seq, RT-PCR should generally prove more sensitive for detecting substantial splicing abnormalities such as exon skipping, since targeted amplification allows a very low limit of detection. However, endpoint RT-PCR and Sanger sequencing are not truly quantitative methods and suffer from biases such as preferential amplification of shorter products. Whole-transcriptome RNA-seq, conversely, may provide more reliable quantification of splice isoforms through calculated read-based metrics such as PSI values.^10^ On the other hand, transcriptome-wide RNA-seq is intrinsically limited in its depth of coverage by the number of reads obtained per sample, particularly where a gene is poorly expressed. A number of RNA-seq samples in this cohort did indeed have relatively poor coverage across the target region for the variants in question. However, where a splice abnormality results in a small-scale change, for example insertion of a few nucleotides as seen with *DKC1* c.915+10G>A, RNA-seq may succeed in identifying this where Sanger sequencing of PCR products fails. Small-scale splicing changes are easily missed on gel electrophoresis and coupled with the poor sensitivity of Sanger sequencing to detect low-level sample heterogeneity, this is an instance where RNA-seq can outperform RT-PCR. Another potential approach to raise coverage depth could be to perform a targeted RNA-seq library prep focused on the gene region of interest. However, this would be at the expense of RNA-seq’s other great advantage: the ability to look for alternative pathogenic splicing events or even alternative pathogenic sequence variants in the same or in other genes.

### Bioinformatic tool comparison

The ability to accurately predict the effect a given sequence variant will have on splicing is highly desirable in prioritizing variants for functional validation, or even as a diagnostic assessment in its own right. However, despite a multitude of different prediction methods being available, there is little consensus on the best tools or the optimal usage and score thresholds to use. A common approach is to score a variant with several (three to five) tools and take a consensus approach—if the majority of tools predict an effect, the variant is predicted to be splice affecting. In our assessment, we found little benefit of this consensus approach over the use of individual tools. Across all scored variants the Alamut 2/3 consensus gave similar sensitivity and specificity to component tools MES and SSF, and gave a comparable AUC in the analysis considering the overlapping variant set that were scored by all tools. The newer, machine learning–based approach, SpliceAI, outperformed the other tools across metrics, classifying over 90% of variants consistently with the experimental data. Our data suggest this method could assist in clinical interpretation of variants potentially affecting splicing, and offer benefits over existing approaches that are currently in use diagnostically.

Despite questions over the accuracy and applicability of in silico splice prediction tools, in this cohort, a high proportion of variants were correctly predicted to alter normal splicing, particularly given the high proportion of variants outside of the immediate splicing area.^14,15^ However, this is likely to be at least partially explained by the bias in case selection, since we expect variants were more likely to be referred for splicing analysis where diagnostic genetic test reports had predicted a possible splicing effect.

### Limitations of testing and using blood as a proxy tissue

In analyzing blood RNA, there are intrinsic limitations. Most obviously, only genes that are transcribed in blood can be detected. Genes that are highly tissue-specific in their expression can therefore prove problematic to analyze. Alternative cell types may be available in some cases from skin or muscle biopsies and RNA from such sources has been successfully used for splicing analysis.^25,26^ However, even in the absence of such samples, low-level basal transcription of the genome is known to take place and some 80% of all human coding sequences have been identified in blood.^27^ In this study, reference was made to GTEx transcript per million (TPM) values (Table S1).^28,29^ Interestingly, informative RT-PCR results were obtainable for a number of genes reported to have TPM values of zero (*FBN2*, *COL3A1*, *COL4A1*, *COL5A1*), although this is not reliably the case for all such genes.

A further important consideration is the tissue specificity of splicing. Use of blood as a proxy tissue assumes that similar splicing events are taking place in clinically relevant tissues, which is not necessarily the case. Another limitation in detection may occur if nonsense-mediated decay (NMD) is efficient enough to remove all abnormally spliced transcripts from a sample. Indeed, variability in NMD contributes to uncertainty in quantifying the relative usage of aberrant splice events.^30,31^ This means that simple quantification metrics of splice site usage are unlikely to be directly informative of pathogenicity and need to be considered in comparison with control samples.

### Mechanistic insights into splicing

**Fig. 4:**
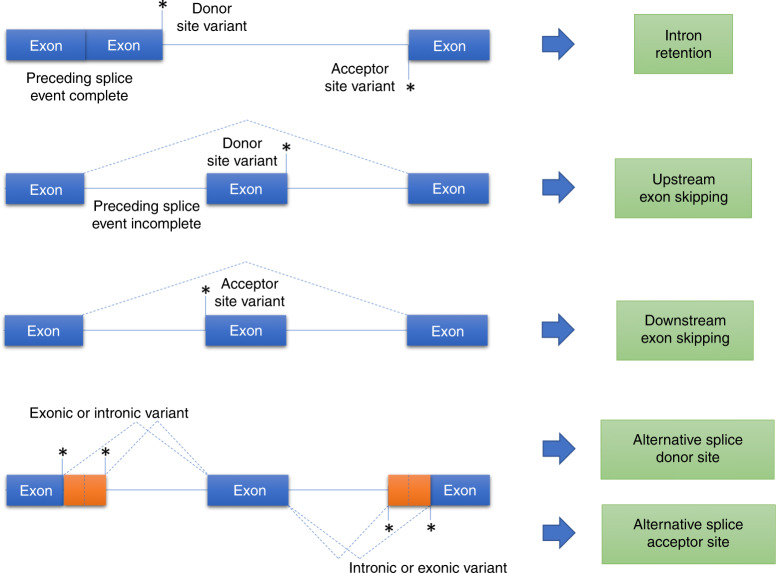
A potential model of splicing disruption. Where an upstream splicing event is complete, a splice donor or acceptor site variant may lead to intron retention. Where a preceding splicing event remains incomplete, a splice donor variant may cause skipping of the upstream exon. Similarly, if a splice acceptor site variant causes an upstream splice donor site to remain unused then this may cause skipping of the exon downstream of the acceptor site variant. Exonic or intronic variants that create or strengthen cryptic splice sites can lead to use of alternative splice donor or acceptor sites.

A notable finding in this study is that splice-altering variants located close to the donor splice site tend to cause skipping of the upstream exon. In considering the splicing reaction, where the donor splice site is first cleaved and ligated to the intronic branch point to form a lariat, one might expect a disrupted donor splice site to cause intron retention. However, retention of introns appears to be a relatively rare event in this study. Furthermore, the presence of upstream exon skipping in these cases implies that splicing of the preceding intron has not yet been completed by the time the next intron is being spliced. If upstream splicing were complete, there would be no upstream donor splice site available to allow exon skipping to take place (Fig. 4), except in the setting of a recursive splicing mechanism.^24^

Splicing is known to occur cotranscriptionally and the choice of splice site depends not only on sequence but also on additional factors such as rate of transcription, RNA secondary structure, chromatin conformation, and the effects of splicing enhancers and silencers.^32^ It may be that some of these factors are playing a role in driving the upstream exon skipping that predominates in this variant cohort. The timing of splicing events may also potentially be influenced somewhat by intron length. However, analysis of the intron–exon structure around these variants did not indicate any significant skewing of upstream versus downstream intron length.

Further work will be needed to better characterize the mechanistic and regulatory elements of the abnormal splicing seen in this study. Understanding the underlying mechanisms governing such splicing abnormalities is critical, not only to allow their better prediction but also to inform therapeutic approaches that aim to correct them. Splice-switching antisense oligonucleotide (ASO) therapies are increasingly being developed for clinical use and their design depends upon accurate targeting of disease-specific splice sites or splice-regulatory elements.^33,34^ The sequence specificity of this approach lends itself ideally to personalized medicine and indeed such a drug has recently been developed for an *N*-of-1 study in a single patient with a deep intronic variant affecting splicing.^35^ In the appropriate disease settings, splice-affecting variants lying within deep intronic or exonic regions therefore represent particularly good candidates for the development of splice-switching ASO therapeutic approaches.

### Conclusion

This variant cohort is among the largest and most diverse to have had experimentally determined RNA splicing effects analyzed and published to date. While routine use of RNA analysis in genetic diagnostics requires further work to clarify the service implications, based on this study, we recommend that RNA-based splicing analysis be at least routinely considered in genetic disease variant interpretation to improve diagnostic uplift. While bioinformatic splicing prediction tools, particularly SpliceAI, continue to improve in accuracy, there is still significant miscalling of predictions from all tools. Ideally, they should therefore not be relied upon in isolation in assessing a variant’s effect on splicing and their predictions should not be a prerequisite line of evidence for classifying splice variants, should clear experimentally obtained RNA splicing evidence be available. Owing to the subtlety and complexity of RNA splicing, additional work will be required to determine how best to incorporate splicing predictions and experimental splicing analysis into variant classification guidelines.

In conclusion, this large study demonstrates the potential of blood RNA analysis in clarifying the effects of variants of unknown significance and the uplift of diagnostic rate.

## Supplementary information

Supplementary Information

Supplementary Figure S1

Supplementary Table S1
