# Correction: Blood RNA analysis can increase clinical diagnostic rate and resolve variants of uncertain significance

**DOI:** 10.1038/s41436-020-0789-2

**Published:** 2020-04-01

**Authors:** Htoo A. Wai, Jenny Lord, Matthew Lyon, Adam Gunning, Hugh Kelly, Penelope Cibin, Eleanor G. Seaby, Kerry Spiers-Fitzgerald, Jed Lye, Sian Ellard, N. Simon Thomas, David J. Bunyan, Andrew G. L. Douglas, Diana Baralle, Splicing and disease working group, Swati Naik, Nicola Ragge, Helen Cox, Jenny Morton, Mary O’Driscoll, Derek Lim, Deborah Osio, Frances Elmslie, Camilla Huber, Julie Hewitt, Heidy Brandon, Meriel McEntagart, Sahar Mansour, Nayana Lahiri, Esther Dempsey, Merrie Manalo, Tessa Homfray, Anand Saggar, Jin Li, Julian Barwell, Kate Chandler, Tracy Briggs, Sofia Douzgou, Julian Adlard, Alison Kraus, Sarju Mehta, Amy Watford, Alan Donaldson, Karen Low, Gabriela Jones, Abhijit Dixit, Elizabeth King, Nora Shannon, Marios Kaliakatsos, Merrie Manalo, Shelagh Joss, Meena Balasubramanian, Diana Johnson, Sarah Everest, Claire Salter, Victoria Harrison, Gillian Wise, Audrey Torokwa, Victoria Sands, Esther Pyle, Tessy Thomas, Katherine Lachlan, Nicola Foulds, Andrew Lotery, Andrew Douglas, Simon Hammans, Emily Pond, Rachel Horton, Mira Kharbanda, David Hunt, Charlene Thomas, Lucy Side, Catherine Willis, Stephanie Greville-Heygate, Rebecca Mawby, Catherine Mercer, Karen Temple, Esther Kinning, Ognjen Bojovic, L. Archer

**Affiliations:** 1 Human Development and Health, Faculty of Medicine, University of Southampton, Southampton, UK; 2 Wessex Regional Genetics Laboratory, Salisbury District Hospital, Salisbury, UK; 3 Exeter Genomics Laboratory, Royal Devon & Exeter NHS Foundation Trust, Exeter, UK; 4 Translational Genomics Unit, Broad Institute of MIT and Harvard, Cambridge, MA USA; 5 Wessex Clinical Genetics Service, University Hospital Southampton NHS Foundation Trust, Southampton, UK

Correction to: *Genetics in Medicine* 2020; 10.1038/s41436-020-0766-9, published online 03 March 2020

In the original version of this Article, the contribution of the Splicing and Disease Working Group was not acknowledged. The PDF and HTML versions of the Article have now been corrected to include the following clinical collaborators:

Swati Naik, Nicola Ragge, Helen Cox, Jenny Morton, Mary O'Driscoll, Derek Lim, Deborah Osio, Frances Elmslie, Camilla Huber, Julie Hewitt; Heidy Brandon, Meriel McEntagart, Sahar Mansour, Nayana Lahiri, Esther Dempsey, Merrie Manalo, Tessa Homfray, Anand Saggar; Jin Li, Julian Barwell; Kate Chandler, Tracy Briggs, Sofia Douzgou, Julian Adlard, Alison Kraus; Sarju Mehta; Amy Watford, Alan Donaldson, Karen Low; Gabriela Jones, Abhijit Dixit, Elizabeth King, Nora Shannon; Marios Kaliakatsos; Merrie Manalo; Shelagh Joss; Meena Balasubramanian, Diana Johnson; Sarah Everest; Claire Salter, Victoria Harrison, Gillian Wise, Audrey Torokwa, Victoria Sands, Esther Pyle, Tessy Thomas, Katherine Lachlan, Nicola Foulds, Andrew Lotery, Andrew Douglas, Simon Hammans, Emily Pond, Rachel Horton, Mira Kharbanda, David Hunt, Charlene Thomas, Lucy Side, Catherine Willis, Stephanie Greville-Heygate, Rebecca Mawby, Catherine Mercer, Karen Temple, Esther Kinning; Ognjen Bojovic; L. Archer.

