# Supplementary material for: Blood RNA analysis can increase clinical diagnostic rate and resolve variants of uncertain significance
Source: Genet Med. 2020 Mar 3;22(6):1005–14. doi: 10.1038/s41436-020-0766-9 (PMC7272326; doi:10.1038/s41436-020-0766-9)
Supplement: Supplementary file 1 — Supplementary Information [file 41436_2020_766_MOESM1_ESM.pdf]

SUPPLEMENTARY INFORMATION

SUPPLEMENTARY METHODS:

Salisbury RNA extraction and RT-PCR protocol

Blood samples were collected into PAXgene RNA collection tubes (Qiagen, UK) and RNA was extracted using the QIAcube Connect extraction machine (Qiagen, UK) and the standard protocol of the RNeasy kit (Qiagen, UK). cDNA preparation was then carried out using 1.5µl of 10mM dNTP mix (Promega, UK), 2µl of 0.1M Dithiothreitol (Invitrogen, UK), 1µl of 40 U/µl Moloney Murine Leukemia Virus Reverse Transcriptase (Invitrogen, UK), 1µl of 40 U/µl RNAaseOut ribonuclease inhibitor (Invitrogen, UK), 4µl of Reverse Transcriptase buffer (Invitrogen, UK), 1µl of 10ng/µl random hexamer primers (Thermo Fisher, UK) and 10µl of RNA. Samples were incubated for one hour at 37°C followed by 10mins at 65°C. For each variant, where possible, RNA analysis was carried out by using a forward PCR primer situated at least two exons upstream from the exon (or flanking intronic sequence) containing the variant and a reverse primer at least two exons downstream from the exon of interest. This was subject to the resultant PCR fragment being of a reasonable size for Sanger sequencing (ideally below about 600 base pairs), and for those genes with relatively small exons the primers were situated further away where possible (all primer sequences available upon request). PCR reactions were carried out in a 20µl volume containing 1.5µl of cDNA, 10nM of each primer (Promega, UK), 2µl of 10x Platinum Taq buffer (Invitrogen, UK), 0.2mM of each dNTP, 1.5mM $MgCl_2$ and 0.5 units of Platinum Taq polymerase (Invitrogen, UK). Cycling parameters were 94°C for 12 minutes followed by 35 cycles of 94°C for 30 seconds, 60°C for 30 seconds and 72°C for 30 seconds. PCR products were checked by gel electrophoresis and then bi-directionally sequenced using the standard protocol of the

Big-Dye® Terminator v1.1 Cycle Sequencing Kit (Applied Biosystems, USA) and separated on an ABI 3130*xl* Genetic Analyzer (Applied Biosystems, USA). Subsequent data were analysed using the Mutation Surveyor (version 3.1) software (SoftGenetics, USA).

Exeter RNA extraction and RT-PCR protocol

RNA was extracted from whole blood (PAXgene Blood RNA tube; Qiagen 762165) on the QIAcube automated nucleotide extraction robot using the PAXgene blood RNA kit (Qiagen 762174) following the manufacturer's protocol. Reverse transcription PCR was done using a random hexamer primer mix and the VILO SuperScript III RT-PCR system (Life Technologies; 11754250) following the manufacturer's protocol. Primers were designed manually using the Primer3Plus software (National Human Genome Research Institute, USA); where possible PCR primers were designed to span exon-exon boundaries. PCR amplification was performed using the Megamix Royal PCR master mix (MicroZone; 2MMR-10). PCR products were visualised by gel electrophoresis (3% agarose) before bi-directional Sanger sequencing. Sanger sequencing was performed using BigDye terminator v3.1 (Applied Biosystems; 4337456) and the Agencourt automated clean-up system (AMPure (Beckman Coulter; A63881), CleanSEQ (Beckman Coulter; A29154)), following the manufacturer's protocol, and sequenced using the ABI 3730 DNA analyser. Sanger sequencing products were visualised using Mutation Surveyor v5.1.2 (SoftGenetics).

Southampton RNA extraction and RT-PCR protocol

Blood was collected in PAXgene Blood RNA tubes (PreAnalytiX, Switzerland). RNA was then extracted from blood samples using PAXgene Blood RNA Kit (PreAnalytiX, Switzerland) and quality control was performed using a 2100 Bioanalyzer instrument (Agilent, UK). RNA extracted from blood samples was converted to cDNA using the High-Capacity cDNA Reverse

Transcription Kit (ThermoFisher Scientific, UK) using random hexamers. Primer pairs were designed manually depending on the genomic locations of variants and ordered from Integrated DNA Technologies (IDT, UK). PCR experiments were performed using GoTaq G2 Polymerase PCR system (Promega, UK) according to the manufacturer's protocol. RT-PCR products were purified by GeneJET PCR Purification Kit (ThermoFisher Scientific, UK) and bidirectional Sanger sequencing was carried out by SourceBioscience (Nottingham, UK). Amplicons were also analysed by agarose gel electrophoresis and imaged by Chemidoc XRS+ (Bio-Rad, USA). Where indicated, amplicons for further analysis were gel-purified by GeneJET Gel Extraction Kit (ThermoFisher Scientific, UK) and cloned into plasmids using a TA cloning kit, with the pCR 2.1 vector (ThermoFisher Scientific, UK). Plasmids carrying inserts were sent to SourceBioscience (Nottingham, UK) for Sanger sequencing.

RNA-seq analysis

QC of read data:

QC was performed on sequencing reads received from Novogene with FastQC (v0.11.3) (https://www.bioinformatics.babraham.ac.uk/projects/fastqc/), and compiled and visualised with MultiQC (v1.5) (https://multiqc.info/).

Alignment with STAR (v2.6.1c)[1]:

The STAR index was created with STAR's genomeGenerate using GRCh38.primary_assembly.genome.fa and gencode.v30.annotation.gtf, both downloaded from GENCODE[2] (https://www.gencodegenes.org/human/) with --sjdbOverhang 149 and all other settings as default. Samples were individually aligned in twopass Basic mode with the following parameters specified, and everything else as default: --outSAMmapqUnique 60, --outFilterType BySJout, --outReadsUnmapped Fastx, --outSAMtype BAM Unsorted.

Samtools[3] (v1.3.2) was used to sort, index and extract regions (using Samtools view) corresponding to the gene harbouring the VOUS.

QC of aligned data:

QC was performed on aligned data using the following components of RSeQC[4] (v2.6.4) (http://rseqc.sourceforge.net/): bam_stat.py, infer_experiment.py, geneBody_coverage.py, junction_annotation.py and junction_saturation.py. Results were compiled and visualised with MultiQC (v1.5) (https://multiqc.info/).

***In silico* splicing predictions**

Equations for sensitivity, specificity, accuracy, positive and negative predictive values:

Sensitivity = true positives / (true positives + false negatives)

Specificity = true negatives / (true negatives + false positives)

Accuracy = (true positives + true negatives) / (all variants)

Positive predictive value = true positives / (true positives + false positives)

Negative predictive value = true negatives / (true negatives + false negatives)

SUPPLEMENTARY FIGURE AND TABLE LEGENDS:

Figure S1. **Variant classifications in ClinVar (as of 13 November 2019).** Proportions and numbers of variants listed in ClinVar under each of the five ACMG classifications.

Table S1. **Tabulated list of variants, splicing effects and bioinformatic predictions.** Variants highlighted in red were found to affect splicing, while blue-highlighted variants were not. SNV position is in reference to the nearest annotated donor (D) or acceptor (A) splice site in the listed transcript, where D-1 is the final nucleotide of an exon and A+1 is the first nucleotide of an exon. Genomic coordinates are listed based on results obtained from the Ensembl Variant Effect Predictor (VEP) downloaded in VCF format.[5] Bioinformatic predictions taken to indicate a splice-altering effect (within applied thresholds) are highlighted in red. For splicing result: IR, intron retention; SE, skipped exon; A5SS, alternative 5´ splice site; A3SS, alternative 3´splice site. For HSF predicted effects: DSB, donor site broken; NDS, new donor site; NAS, new acceptor site; ASB, acceptor site broken; "no result" refers to where HSF made erroneous calls of input sequence elements, leading to inappropriate predictions. For SSF, MaxEntScan, NNSPLICE: NSS, native splice site. SpliceAI values were obtained from a pre-computed score file (v1.3). *These two *MKKS* variants were present *in trans* in a single patient. **This sample contained two separate but closely linked monoallelic *NF1* variants, which have been considered together as a single variant for the purposes of this study.

REFERENCES

1. Dobin A, Davis CA, Schlesinger F, et al. STAR: Ultrafast universal RNA-seq aligner. *Bioinformatics*. 2013;29(1):15-21. doi:10.1093/bioinformatics/bts635
2. Frankish A, Diekhans M, Ferreira AM, et al. GENCODE reference annotation for the human and mouse genomes. *Nucleic Acids Res*. 2019;47(D1):D766-D773. doi:10.1093/nar/gky955
3. Li H, Handsaker B, Wysoker A, et al. The Sequence Alignment/Map format and SAMtools. *Bioinformatics*. 2009;25(16):2078-2079. doi:10.1093/bioinformatics/btp352
4. Wang L, Wang S, Li W. RSeQC: quality control of RNA-seq experiments. *Bioinformatics*. 2012;28(16):2184-2185. doi:10.1093/bioinformatics/bts356
5. McLaren W, Gil L, Hunt SE, et al. The Ensembl Variant Effect Predictor. *Genome Biol*. 2016;17(1):122. doi:10.1186/s13059-016-0974-4
