# Supplementary figures and images for: Blood RNA analysis can increase clinical diagnostic rate and resolve variants of uncertain significance
Source: Genet Med. 2020 Mar 3;22(6):1005–14. doi: 10.1038/s41436-020-0766-9 (PMC7272326; doi:10.1038/s41436-020-0766-9)

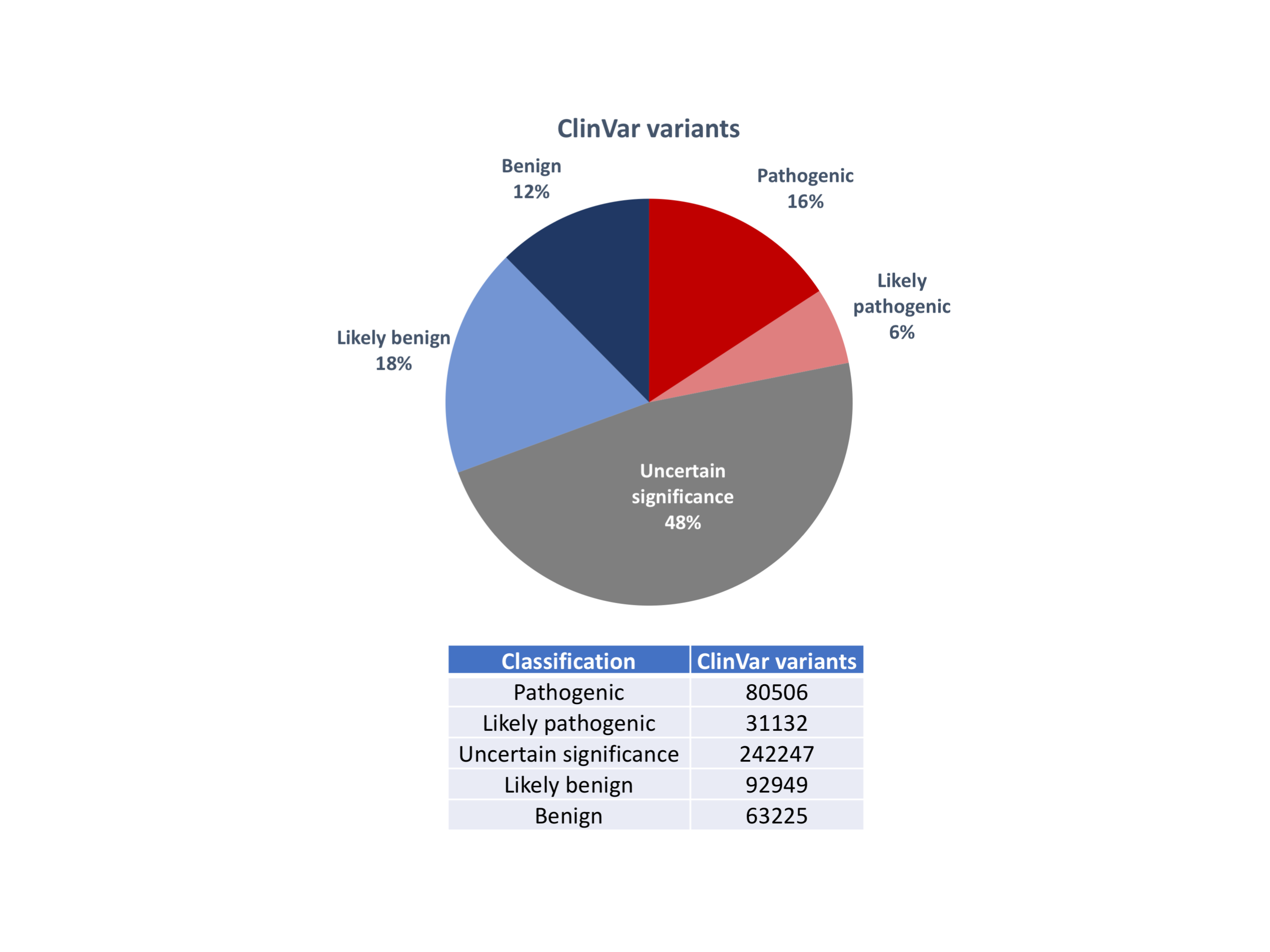

Supplement: Supplementary file 2 — Supplementary Figure S1 [file 41436_2020_766_MOESM2_ESM.tiff]
